# Clinical characteristics and post‐operative outcomes in children with malformation of cortical development related drug‐resistant epilepsy: 428 cases in one pediatric epilepsy center

**DOI:** 10.1111/cns.70031

**Published:** 2024-09-04

**Authors:** Hao Yu, Yu Sun, Chang Liu, Yao Wang, Qingzhu Liu, Taoyun Ji, Shuang Wang, Xiaoyan Liu, Yuwu Jiang, Lixin Cai

**Affiliations:** ^1^ Pediatric Epilepsy Center Peking University First Hospital Beijing China

**Keywords:** children, different age, drug‐resistant epilepsy, malformation of cortical development, surgery

## Abstract

**Aims:**

To investigate post‐operative seizure outcomes, and predictors of surgical outcomes of the malformation of cortical development (MCD) in children with drug‐resistant epilepsy (DRE) and age‐specific characteristics.

**Methods:**

We retrospectively analyzed clinical data from 428 children with MCD‐related DRE who underwent curative surgical treatment. Statistical analyses were conducted to identify correlative characteristics, prognostic predictors, and differences among various age groups.

**Results:**

After more than 3 years of follow‐up, 81.3% of patients achieved Engel I outcomes. Prognosis was correlated with factors such as age at surgery, MRI findings, invasive EEG, pathology, acute postoperative seizures (APOS), and the number of preoperative and postoperative anti‐seizure medications (AEDs). Age at surgery and the number of preoperative AEDs (*p* < 0.001) were significant predictors of seizure recurrence. Distinct clinical characteristics were observed among different age groups.

**Conclusion:**

Surgery is effective in terminating MCD‐related DRE. Younger age at surgery and fewer preoperative AEDs are associated with better prognoses. Clinical characteristics vary significantly with age.

## Graphical abstract

Surgery for malformation of cortical development (MCD) in children with drug‐resistant epilepsy shows 81.3% success. Early surgery, fewer pre‐op anti‐seizure medications predict better outcomes. Different ages in MCD patients show varied clinical traits.

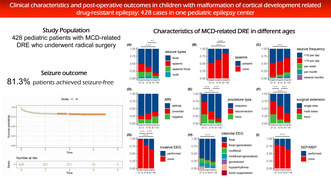

## 1 INTRODUCTION

Malformation of Cortical Development (MCD) is a primary etiological factor in pediatric cases of drug‐resistant epilepsy (DRE) that often requires surgical intervention.^1^ MCD predominantly arises from somatic mutations during embryonic neurodevelopment^2^ leading to refractory seizures, developmental anomalies, and potential epileptic encephalopathy.^3^, ^4^ Surgical resection of the aberrant cortical regions in MCD has been proven effective in controlling seizures, enhancing developmental trajectories, and mitigating adverse effects associated with antiepileptic drugs, thus advocating for early surgical intervention. This highlights the importance of timely diagnosis and targeted therapeutic strategies in the management of MCD‐related epilepsy.^5^

The incidence of epilepsy demonstrates significant age‐dependent variability, with a notably higher occurrence in pediatric populations, particularly among younger children, compared to other age groups.^6^, ^7^, ^8^ This variation is further accentuated by distinct clinical manifestations of epilepsy in young children, which diverge significantly from those observed in other demographic cohorts.^9^ Consequently, there has been a growing interest in clinical research focused on surgical interventions tailored for pediatric patients. Moreover, children across different age brackets exhibit unique anatomical and clinical profiles, necessitating age‐specific modifications in both diagnostic and therapeutic approaches.

In a seminal study, Cloppenborg conducted a retrospective comparative analysis of clinical features in pediatric versus adult populations undergoing surgical treatment for DRE over an extensive period exceeding two decades. This research highlighted the diverse etiological factors in pediatric cases, with generally more favorable outcomes compared to adult counterparts.^10^ The heterogeneous nature of DRE in children, attributable to the developmental stage of their nervous system, is characterized by variable structural^11^ and electroencephalographic (EEG)^12^ features, which critically influence surgical strategies.

Despite these advancements, a conspicuous gap remains in the literature regarding the specific characteristics of MCD‐related DRE across different pediatric age groups. To address this gap, the present study retrospectively collated cases of pediatric DRE associated with MCD from a single center. It undertook a comprehensive comparative analysis of surgical outcomes and clinical features among varying age groups, aiming to elucidate age‐specific considerations in the management of MCD‐related DRE.

## 2 MATERIALS AND METHODS

### 2.1 Inclusion criteria of patients

This study included 428 consecutive pediatric patients diagnosed with DRE associated with MCD, who underwent radical epilepsy surgery at the Pediatric Epilepsy Center, Peking University First Hospital, between January 2017 and December 2020. Inclusion criteria were: (a) diagnosis of DRE according to the International League Against Epilepsy (ILAE) criteria,^13^ or frequent seizures with identifiable structural epileptogenic lesions despite a shorter duration of epilepsy; (b) comprehensive preoperative evaluation supporting candidacy for craniotomy aimed at radical resection or disconnection of the epileptogenic lesion; (c) minimum postoperative follow‐up duration of 3 years; (d) confirmation of MCD through postoperative pathological examination.

### 2.2 Pre‐surgery examinations

Prior to surgical intervention, all participants underwent a comprehensive preoperative assessment. This included long‐term scalp video electroencephalography (VEEG) utilizing the standard 10–20 system protocol. Additionally, patients were subjected to three‐dimensional high‐resolution magnetic resonance imaging (MRI; 3.0 Tesla), which encompassed T1‐weighted, T2‐weighted, Fluid‐attenuated inversion recovery (FLAIR), and diffusion‐weighted imaging (DWI) sequences. Fluorodeoxyglucose positron emission tomography (FDG‐PET) was employed to profile cerebral metabolism.

To enhance precision in delineating the extent of resection or disconnection, MRI‐PET fusion was performed using Sinovation software (Beijing Sinovation Medical Technology Co., Ltd.). This approach was particularly critical in cases where the epileptogenic lesion's location or extent presented significant challenges. For patients where the lesion's location or extent was difficult to determine, systematic invasive EEG examinations were planned. The determination of the surgical strategy, specifically the radical resection of the lesion, was grounded in the insights obtained from these invasive EEG results.

Interictal EEG recordings were classified based on the presence and distribution of epileptiform discharges. The classifications used were: (1) focal, indicating localized epileptiform discharges; (2) multifocal, indicating multiple independent epileptiform foci; (3) generalized, indicating widespread bilateral synchronous discharges; (4) focal + generalized, indicating a combination of localized and generalized discharges; (5) hypsarrhythmia; and (6) burst‐suppression.

In this study, MRI assessments of all participants were rigorously evaluated by three senior radiologists, each with over several years of experience in pediatric neuroimaging. This evaluation facilitated the categorization of patients into three distinct lesion detectability groups: (1) ‘Definite’, characterizing cases where lesions were conspicuously visible; (2) ‘Uncertain’, referring to instances with indeterminate lesion visibility; (3) ‘Negative’, indicating scenarios where lesions were not detectable.

Moreover, the seizure manifestations observed within the cohort were methodically classified into four distinct phenotypic groups: (1) focal; (2) spasms; (3) focal + spasms (with only one type of focal seizure and spasms); (4) multiple types of seizures (more than one type of seizure, excluding the previous category).

Pathological examination adhered stringently to the latest criteria set forth by the International League Against Epilepsy (ILAE) in 2022.^14^ Significantly, the recent introduction of Mild Malformation with Oligodendroglial Hyperplasia in Epilepsy (MOGHE) into the classification necessitated a comprehensive reevaluation of all MCD diagnoses in the study. This led to the reclassification of patients into five distinct categories, as systematically outlined in Table 1.

**TABLE 1:** Clinic characteristics and univariate analysis for seizure outcome.

	[All]	ENGEL I	ENGEL II‐IV	*p*.overall
	*N* = 428	*N* = 368	*N* = 60	
Surgery age (years)	3.82 [2.25;6.68]	3.72 [2.19;6.37]	4.79 [2.71;8.46]	0.040
Onset age (years)	0.67 [0.24;2.53]	0.66 [0.23;2.19]	1.02 [0.32;3.01]	0.285
Duration	2.41 [1.47;4.05]	2.35 [1.47;3.86]	3.17 [1.60;5.40]	0.070
Sex				
Male	273 (63.8%)	234 (63.6%)	39 (65.0%)	0.947
Female	155 (36.2%)	134 (36.4%)	21 (35.0%)	
MRI				
Definite	287 (67.1%)	257 (69.8%)	30 (50.0%)	0.008
Uncertain	67 (15.7%)	54 (14.7%)	13 (21.7%)	
Negative	74 (17.3%)	57 (15.5%)	17 (28.3%)	
Interictal EEG				
Focal	298 (69.6%)	258 (70.1%)	40 (66.7%)	0.484
Focal + generalized	9 (2.10%)	8 (2.17%)	1 (1.67%)	
Multifocal	54 (12.6%)	44 (12.0%)	10 (16.7%)	
Multifocal + generalized	50 (11.7%)	45 (12.2%)	5 (8.33%)	
Generalized	2 (0.47%)	2 (0.54%)	0 (0.00%)	
Hypsarrhythmia	13 (3.04%)	9 (2.45%)	4 (6.67%)	
Burst‐suppression	2 (0.47%)	2 (0.54%)	0 (0.00%)	
Seizure types				
Focal	270 (63.1%)	233 (63.3%)	37 (61.7%)	0.752
Spasms	81 (18.9%)	67 (18.2%)	14 (23.3%)	
Spasms + focal	53 (12.4%)	46 (12.5%)	7 (11.7%)	
Multitypes	24 (5.61%)	22 (5.98%)	2 (3.33%)	
Spasms				
Present	148 (34.6%)	125 (34.0%)	23 (38.3%)	0.608
None	280 (65.4%)	243 (66.0%)	37 (61.7%)	
Seizure frequency				
>10 per day	176 (41.1%)	153 (41.6%)	23 (38.3%)	0.946
<10 per day	199 (46.5%)	169 (45.9%)	30 (50.0%)	
Per week	31 (7.24%)	26 (7.07%)	5 (8.33%)	
Per month	13 (3.04%)	12 (3.26%)	1 (1.67%)	
Several months	9 (2.10%)	8 (2.17%)	1 (1.67%)	
Side				
L	215 (50.2%)	182 (49.5%)	33 (55.0%)	0.511
R	213 (49.8%)	186 (50.5%)	27 (45.0%)	
Invasive EEG				
Performed	78 (18.2%)	60 (16.3%)	18 (30.0%)	0.018
None	350 (81.8%)	308 (83.7%)	42 (70.0%)	
SEP/MEP				
Performed	112 (26.2%)	92 (25.0%)	20 (33.3%)	0.229
None	316 (73.8%)	276 (75.0%)	40 (66.7%)	
Procedure types				
Resection	230 (53.7%)	194 (52.7%)	36 (60.0%)	0.574
Disconnection	122 (28.5%)	107 (29.1%)	15 (25.0%)	
Hemispherotomy	76 (17.8%)	67 (18.2%)	9 (15.0%)	
Surgical extension				
Single lobe	181 (42.3%)	159 (43.2%)	22 (36.7%)	0.359
Multilobes	171 (40.0%)	142 (38.6%)	29 (48.3%)	
Hemisphere	76 (17.8%)	67 (18.2%)	9 (15.0%)	
Pathology				
FCD1	80 (18.7%)	66 (17.9%)	14 (23.3%)	0.004
FCD2	205 (47.9%)	188 (51.1%)	17 (28.3%)	
FCD3	23 (5.37%)	21 (5.71%)	2 (3.33%)	
MOGHE	14 (3.27%)	12 (3.26%)	2 (3.33%)	
Other MCDs	106 (24.8%)	81 (22.0%)	25 (41.7%)	
APOS				
Present	64 (15.0%)	47 (12.8%)	17 (28.3%)	0.003
None	364 (85.0%)	321 (87.2%)	43 (71.7%)	
Habitual type				
Present	18 (4.21%)	12 (3.26%)	6 (10.0%)	0.028
None	410 (95.8%)	356 (96.7%)	54 (90.0%)	
Presurgical AEDs	2.68 (0.95)	2.62 (0.94)	3.02 (0.95)	0.004
Postsurgical AEDs	1.24 (1.07)	1.02 (0.94)	2.57 (0.85)	<0.001

*Note*: For continuous variables, quartiles are represented in brackets, and the Wilcoxon rank‐sum test is used for statistical analysis. For categorical variables, percentages are given in parentheses, and Pearson's chi‐squared test or Fisher's exact test is used. A *p*‐value of <0.05 is considered to indicate statistical significance.

Abbreviations: APOS, acute postoperative seizure; EEG, electroencephalogram; FCD, focal cortical dysplasia; MCD, malformation of cortical development; MOGHE, mild malformation with oligodendroglial hyperplasia in epilepsy; MRI, magnetic resonance imaging; postsurgical AEDs, postsurgical number of anti‐seizure medicines; presurgical AEDs, presurgical number of anti‐seizure medicines; SEP/MEP, somatosensory evoked potentials/motor evoked potentials.

For succinctness and clarity, the criteria for the classification of other study variables were exhaustively itemized in Table 1 and were not elaborated further in the text.

### 2.3 Surgery procedure

During the surgical procedures, intraoperative electrocorticography (ECoG) was systematically utilized to enhance the localization of the epileptogenic zone (EZ). This advanced neurophysiological technique facilitated real‐time refinement of the initially planned surgical strategy, tailoring it to the specific intraoperative findings. Particularly in cases where the epileptogenic lesion encompassed the sensory‐motor cortex, additional neurophysiological assessments, including motor‐evoked potentials (MEP) and somatosensory‐evoked potentials (SEP), were meticulously conducted. These evaluations were critical for preserving essential cortical functions while achieving maximal lesion resection.

To ensure consistency and expertise in surgical procedures, all operations were conducted by a singular, highly experienced neurosurgical team, led by a seasoned neurosurgeon. This approach was adopted to maintain uniformity in surgical techniques and optimize patient outcomes, given the complex nature of these interventions.

### 2.4 Seizure outcome

The assessment of seizure outcomes post‐surgery was rigorously conducted using the Engel classification system.^15^ In this study, seizures manifesting within the first week following surgery were classified as acute postoperative seizures (APOS).^16^ This classification was critical for distinguishing between immediate post‐surgical seizure activity and longer‐term outcomes.

In the application of Kaplan–Meier survival analysis methodologies, the occurrence of seizures at the final follow‐up point was a key consideration. For cases where seizures persisted at the last follow‐up, the onset of the first postoperative seizure was identified as the point of seizure recurrence. Conversely, in scenarios where the last follow‐up was seizure‐free, yet seizures manifested subsequent to the first postoperative week, the occurrence of the initial postoperative seizure was marked as the endpoint for survival analysis purposes.

The protocol for the discontinuation of antiseizure medication (ASM) was carefully designed, typically initiating not earlier than 2 years following the surgical intervention.

Consistent with the study's rigorous follow‐up regimen, routine scalp VEEG examinations were systematically scheduled. These evaluations were conducted at critical postoperative milestones: 3 months, 1 year, and then annually, to monitor the patient's neurophysiological status and to detect any potential recurrence of epileptic activity.

Additionally, a comprehensive postoperative MRI assessment was conducted at the 3‐month mark. This imaging was essential for evaluating intracranial conditions post‐surgery. Key aspects assessed included the presence of brain hydrocephalus, the completeness of the epileptogenic lesion resection, or the adequacy of any surgical disconnection performed.

### 2.5 Clinical characteristics of different age groups

In this study, patients were categorized into three age groups for surgery: 0–3 years, 3–6 years, and 6–18 years. Comparative analysis of these groups focused on assessing their distinct clinical characteristics and surgical outcomes.

### 2.6 Ethics and informed consent

This investigation was approved by the Ethics Committee of Peking University First Hospital. Written consent was obtained from the parents of each participant for the use of their children's data for research purposes.

### 2.7 Statistical analysis

In this study, the Shapiro–Wilk test was employed to ascertain the normality of data distribution. For continuous variables demonstrating a skewed distribution, the Wilcoxon rank‐sum test was utilized for univariate analysis of prognostic factors. Conversely, categorical variables were analyzed using Pearson's chi‐squared test or Fisher's exact test, as appropriate. Subsequently, variables exhibiting a significance level below 0.05 in the preliminary univariate analysis were further examined through multivariate logistic regression analysis.

Additionally, the Kaplan–Meier survival curve was instrumental in estimating the probability of sustained seizure freedom over time. The log‐rank test was applied to evaluate the association between variables with notable differences in the univariate analysis and the incidence of seizure recurrence. All statistical analyses were conducted using R version 4.3.1 (Lucent Technologies, New Jersey, USA).

## 3 RESULTS

### 3.1 Clinical characteristics and presurgical workups

This study included a total of 428 patients who met the inclusion criteria, comprising 273 males and 155 females. The median age at surgery was 3.82 years, with a median age of onset 0.67 years, and a median disease duration of 2.41 years (numbers in brackets following continuous variables in Table 1 represent quartiles). The average number of AEDs taken before surgery was 2.68, and at the last follow‐up, the average number of AEDs was 1.24.

The age groups (0–3 years, 3–6 years, and 6–18 years) were defined based on significant developmental stages and clinical practice in pediatric neurology. The 0–3 years group captures early childhood development, which is a critical period for brain growth and seizure activity. The 3–6 years group corresponds to preschool age, during which further neurological and cognitive developments occur. The 6–18 years group encompasses school‐age children and adolescents, a period marked by more stable neurological development.

Preoperative MRI revealed clear structural abnormalities in 287 patients (63.8%), suspected structural abnormalities in 67 patients (15.7%), and negative structural abnormalities in 74 patients (17.3%).

Among the patients, 176 (41.1%) experienced seizures more than 10 times a day, 199 (46.5%) had seizures daily but fewer than 10 times, and 31 (7.24%) had weekly seizures every. Seizures occurring once a month and once every few months were reported by13 (3.04%) and 9 (2.10%) patients, respectively. Overall, more than 85% of the children experienced daily seizures. Symptomatically, 270 children (63.1%) had only one type of focal non‐spasm seizure, while 81 children (18.9%) exclusively had spasms. There were 53 children (12.4%) with both spasms and another type of focal seizure. Additionally, 24 children (5.61%) had two or more types of seizures, with or without spasms. In summary, 148 children (34.6%) experienced spasms.

Regarding interictal EEG findings, 298 cases (69.6%) showed only focal epileptogenic discharges, while the remaining children exhibited multifocal or generalized epileptogenic discharges (Table 1).

### 3.2 Surgery, pathology and complications

Of the 428 patients, 215 (50.2%) underwent surgery on the left hemisphere, and 213 (49.8%) on the right hemisphere. Seventy‐eight patients (18.2%) underwent preoperative invasive EEG, including sub‐dural and stereoelectroencephalography (SEEG). Intraoperative SEP/MEP was performed on 112 patients (26.2%). The surgical approaches included resection in (230 cases, 53.7%), disconnection (122 cases, 28.5%), and hemispheric disconnection (76 cases, 17.8%). Postoperative pathology results showed FCD2 as the most prevalent diagnosis (205 cases, 47.9%), followed by other MCDs (106 cases, 24.8%).

Five patients had a history of previous radical epilepsy surgery at other hospitals before undergoing surgery at our center. Two patients had undergone Vagus Nerve Stimulation (VNS), and one had undergone stereoelectroencephalography (SEEG) radiofrequency ablation at an external institution preoperatively. All experienced postoperative seizures, leading them to seek further treatment at our hospital.

Twenty‐nine patients underwent a second or third surgery, including four children who underwent two radical epilepsy lesion resection surgeries after the initial procedure. One patient developed hydrocephalus postoperatively and underwent ventriculoperitoneal shunt surgery without any further complications.

### 3.3 Seizure outcome and correlation with clinical characteristics

**FIGURE 1:**
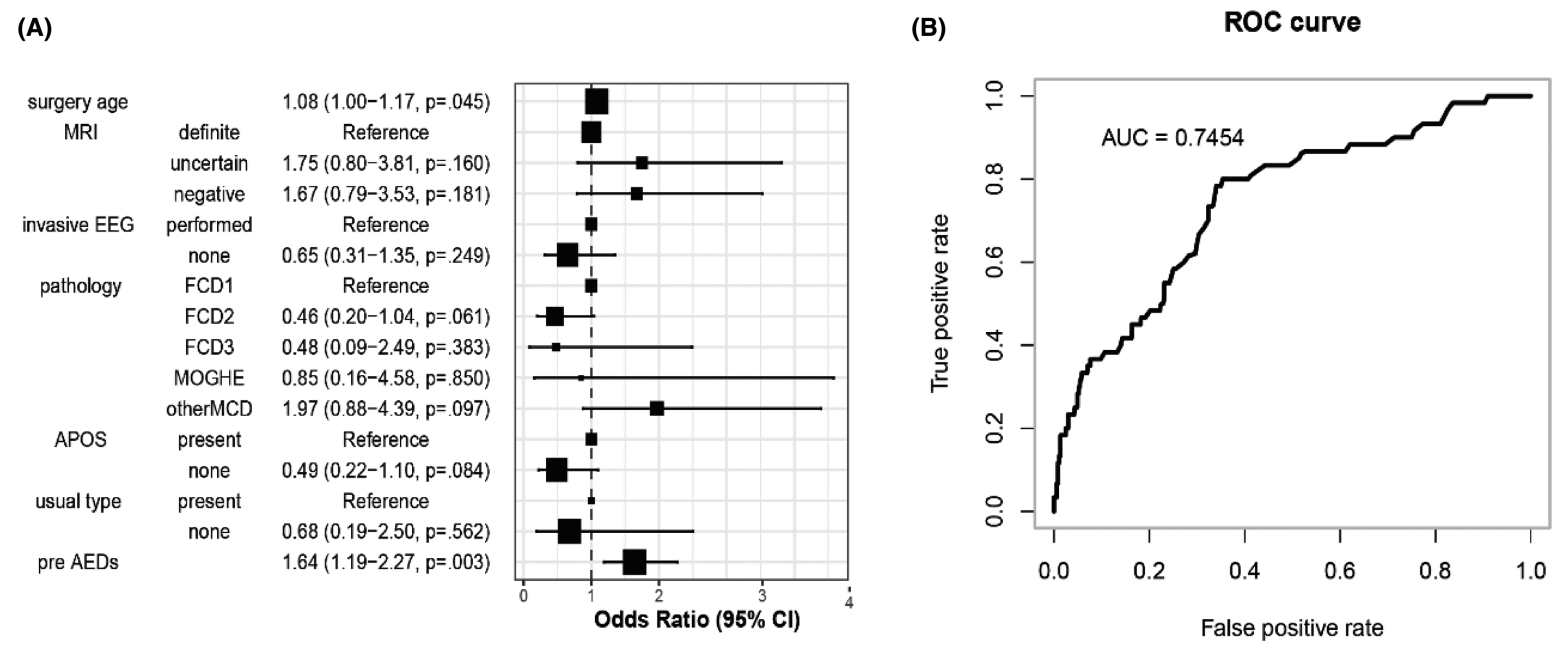
(A) Multivariate logistic regression analysis: Surgery age (*p* < 0.05) and presurgical number of anti‐seizure medicines (AEDs) (*p* < 0.001) was a predictor of seizure recurrence; (B) Receiver operation characteristics (ROC) curve of the logistics model and area under curve (AUC) = 0.7454.

In the univariable correlation analysis, surgery age, MRI findings, invasive EEG use, pathology results, pre‐ and postoperative AEDs counts, APOS, and habitual seizure type after surgery were all correlated with prognosis (*p* < 0.05). Among these prognosis‐related variables, except for postoperative AEDs count, the others were included in multivariate logistic regression analysis. The results revealed that surgery age and preoperative AEDs count were significant prognostic factors for surgical treatment of MCD (*p* < 0.05) (Figure 1A). Specifically, older age at surgery was associated with poorer epilepsy prognosis; and a greater number of preoperative AEDs predicted worse epilepsy prognosis.

Using receiver operation characteristics (ROC) curve analysis to evaluate the predictive ability of the logistic regression model yielded an area under curve (AUC) of 0.7454, indicating a satisfactory performance (Figure 1B).

**FIGURE 2:**
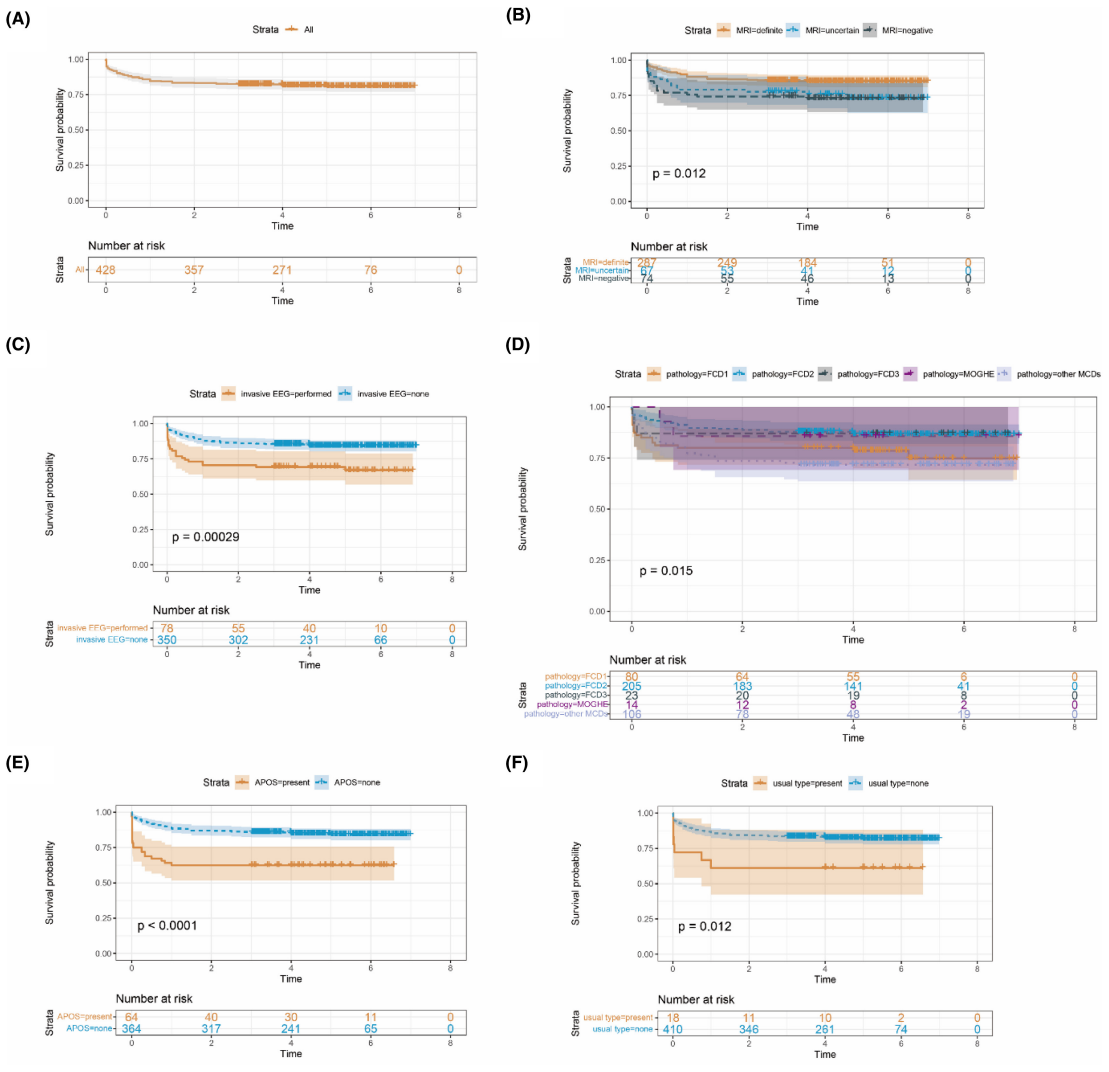
(A) Survival analysis illustrating the chances of postsurgical seizure freedom; (B–E) The log‐rank test demonstrated statistical differences for (B) MRI, (C) invasive EEG, (D) pathology, (E) APOS, and (F) habitual type seizure in perioperative period (*p* < 0.01).

The Kaplan–Meier curve demonstrated that after at least 3 years of follow‐up, 81.3% of the children were seizure‐free (Figure 2A). Among the 29 patients who continued to experience seizures following the initial surgery, 21 achieved seizure‐free status at the last follow‐up after undergoing second or third surgeries, resulting in a seizure‐free rate of 72.4% after multiple surgeries (Table 1 in Supplementary). Additionally, 7 patients achieved seizure‐free status through medication adjustment, increasing the seizure‐free rate at the last follow‐up to 86.0%.

A log‐rank test revealed statistically significant differences in seizure prognosis based on MRI findings, invasive EEG use, pathology results, APOS, and habitual seizure type after surgery. This result aligned with the findings of the univariate analysis (*p* < 0.01) (Figure 2B–E).

### 3.4 Characteristics among different age groups

**FIGURE 3:**
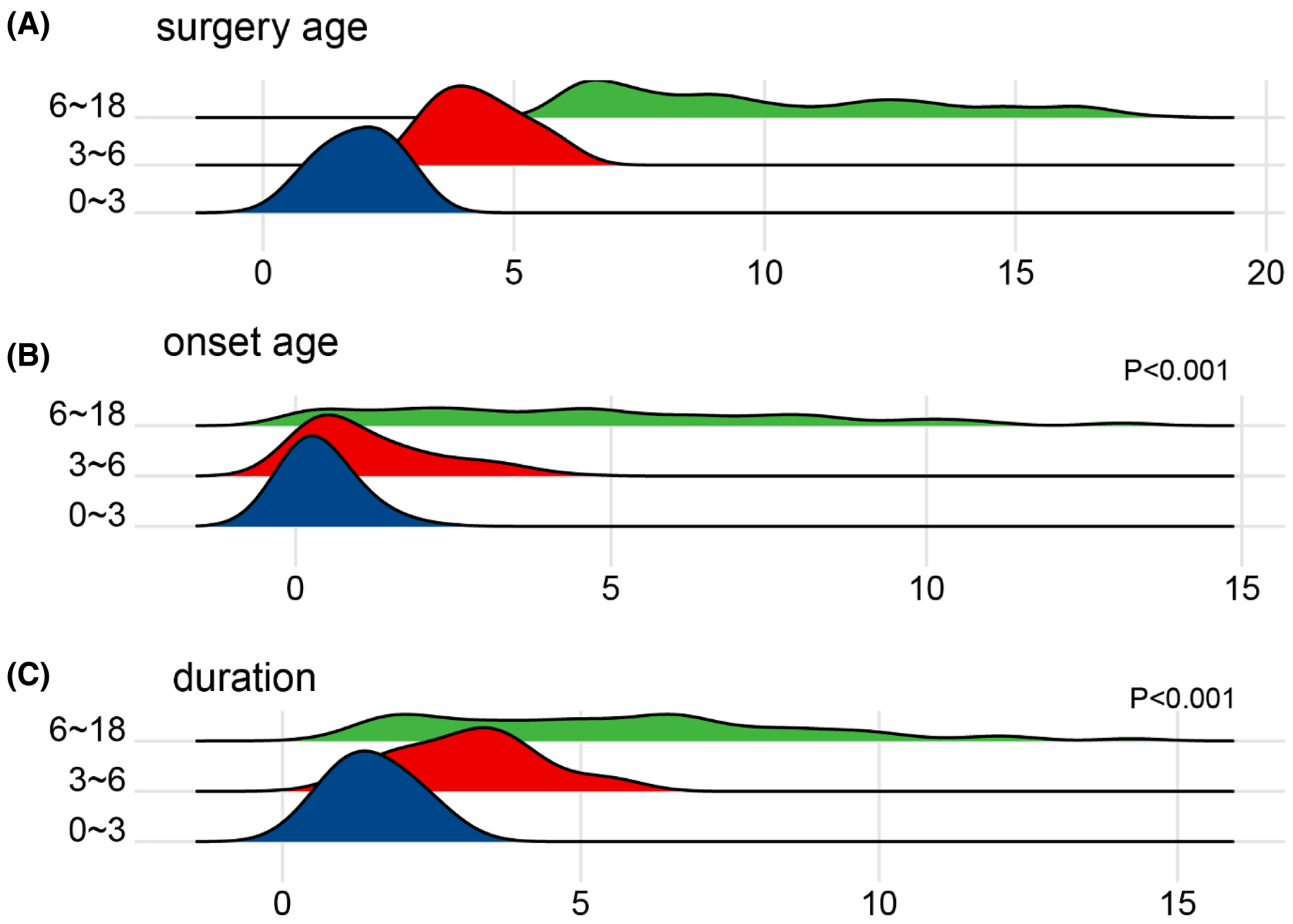
Surgery age (A), onset age (B), and seizure duration (C) in different age groups showing onset age and seizure duration in different age groups differs significantly.

The onset age and epilepsy duration differed significantly among different age groups, with younger surgical ages associated with earlier onset and shorter duration of epilepsy (Figure 3).

In terms of structural abnormalities on MRI, a greater proportion of older individuals exhibited suspected or negative lesions (*p* = 0.046). Regarding seizure types, younger age was associated with a higher prevalence of spasms or various types of seizures (*p* < 0.001). In terms of surgical strategy, invasive EEG (*p* < 0.001) and SEP/MEP (*p* = 0.010) were more frequently employed in older patients. Concerning the surgical approach and extension, younger age was associated with a higher utilization of unilobar or multilobar disconnection surgery or hemispherotomy (*p* < 0.001), and a larger extent of surgical resection or disconnection (*p* < 0.001). Among patients with postoperative habitual type seizures, a higher proportion was observed in older individuals (*p* = 0.015). However, there was no significant difference in prognosis among patients in three different age groups, although a difference existed in the correlation between age as a continuous variability and seizure outcomes in univariate analysis (Table 2).

**TABLE 2:** Clinic characteristics of different age groups.

	[All]	0 ~ 3 years	3 ~ 6 years	6 ~ 18 years	*p*.overall
	*N* = 428	*N* = 166	*N* = 132	*N* = 130	
Onset age (years)	0.67 [0.24;2.53]	0.25 [0.07;0.52]	0.76 [0.38;1.74]	4.32 [1.96;6.95]	<0.001
Duration (years)	2.41 [1.47;4.05]	1.40 [1.05;2.04]	3.23 [2.25;3.78]	5.31 [2.97;7.27]	<0.001
Sex					
Male	273 (63.8%)	102 (61.4%)	84 (63.6%)	87 (66.9%)	0.622
Female	155 (36.2%)	64 (38.6%)	48 (36.4%)	43 (33.1%)	
MRI					
Definite	287 (67.1%)	121 (72.9%)	87 (65.9%)	79 (60.8%)	0.046
Uncertain	67 (15.7%)	20 (12.0%)	27 (20.5%)	20 (15.4%)	
Negative	74 (17.3%)	25 (15.1%)	18 (13.6%)	31 (23.8%)	
Interictal EEG					
Focal	298 (69.6%)	109 (65.7%)	93 (70.5%)	96 (73.8%)	0.026
Focal+generalized	9 (2.10%)	1 (0.60%)	2 (1.52%)	6 (4.62%)	
Multifocal	54 (12.6%)	25 (15.1%)	15 (11.4%)	14 (10.8%)	
Multifocal + generalized	50 (11.7%)	20 (12.0%)	19 (14.4%)	11 (8.46%)	
Generalized	2 (0.47%)	0 (0.00%)	0 (0.00%)	2 (1.54%)	
Hypsarrhythmia	13 (3.04%)	9 (5.42%)	3 (2.27%)	1 (0.77%)	
Burst‐suppression	2 (0.47%)	2 (1.20%)	0 (0.00%)	0 (0.00%)	
Seizure types					
Focal	270 (63.1%)	85 (51.2%)	80 (60.6%)	105 (80.8%)	<0.001
Spasms	81 (18.9%)	44 (26.5%)	23 (17.4%)	14 (10.8%)	
Spasms + focal	53 (12.4%)	29 (17.5%)	19 (14.4%)	5 (3.85%)	
Multitypes	24 (5.61%)	8 (4.82%)	10 (7.58%)	6 (4.62%)	
Spasms					
Present	148 (34.6%)	78 (47.0%)	49 (37.1%)	21 (16.2%)	<0.001
None	280 (65.4%)	88 (53.0%)	83 (62.9%)	109 (83.8%)	
Seizure frequency					
>10 per day	176 (41.1%)	79 (47.6%)	56 (42.4%)	41 (31.5%)	0.057
<10 per day	199 (46.5%)	78 (47.0%)	59 (44.7%)	62 (47.7%)	
Per week	31 (7.24%)	7 (4.22%)	10 (7.58%)	14 (10.8%)	
Per month	13 (3.04%)	2 (1.20%)	5 (3.79%)	6 (4.62%)	
Several months	9 (2.10%)	0 (0.00%)	2 (1.52%)	7 (5.38%)	
Side					
L	215 (50.2%)	91 (54.8%)	70 (53.0%)	54 (41.5%)	0.057
R	213 (49.8%)	75 (45.2%)	62 (47.0%)	76 (58.5%)	
Invasive EEG					
Performed	78 (18.2%)	4 (2.41%)	27 (20.5%)	47 (36.2%)	<0.001
None	350 (81.8%)	162 (97.6%)	105 (79.5%)	83 (63.8%)	
SEP/MEP					
Performed	112 (26.2%)	33 (19.9%)	33 (25.0%)	46 (35.4%)	0.010
None	316 (73.8%)	133 (80.1%)	99 (75.0%)	84 (64.6%)	
Procedure type					
Resection	230 (53.7%)	46 (27.7%)	77 (58.3%)	107 (82.3%)	<0.001
Disconnection	122 (28.5%)	69 (41.6%)	37 (28.0%)	16 (12.3%)	
Hemispherotomy	76 (17.8%)	51 (30.7%)	18 (13.6%)	7 (5.38%)	
Surgical extension					
Single lobe	181 (42.3%)	41 (24.7%)	56 (42.4%)	84 (64.6%)	<0.001
Multilobes	171 (40.0%)	74 (44.6%)	58 (43.9%)	39 (30.0%)	
Hemisphere	76 (17.8%)	51 (30.7%)	18 (13.6%)	7 (5.38%)	
Pathology					
FCD1	80 (18.7%)	31 (18.7%)	27 (20.5%)	22 (16.9%)	0.058
FCD2	205 (47.9%)	78 (47.0%)	58 (43.9%)	69 (53.1%)	
FCD3	23 (5.37%)	5 (3.01%)	5 (3.79%)	13 (10.0%)	
MOGHE	14 (3.27%)	7 (4.22%)	6 (4.55%)	1 (0.77%)	
Other MCDs	106 (24.8%)	45 (27.1%)	36 (27.3%)	25 (19.2%)	
APOS					
Present	64 (15.0%)	28 (16.9%)	15 (11.4%)	21 (16.2%)	0.375
None	364 (85.0%)	138 (83.1%)	117 (88.6%)	109 (83.8%)	
Habitual type					
Present	18 (4.21%)	4 (2.41%)	3 (2.27%)	11 (8.46%)	0.015
None	410 (95.8%)	162 (97.6%)	129 (97.7%)	119 (91.5%)	
pre_AEDs	2.68 (0.95)	2.62 (0.94)	2.74 (1.05)	2.68 (0.85)	0.544
post_AEDs	1.24 (1.07)	1.10 (1.06)	1.37 (1.06)	1.29 (1.09)	0.072
LFU					
I	368 (86.0%)	147 (88.6%)	117 (88.6%)	104 (80.0%)	0.063
II–IV	60 (14.0%)	19 (11.4%)	15 (11.4%)	26 (20.0%)	

*Note*: For continuous variables, quartiles are provided in brackets, and the statistical method used is the Wilcoxon rank‐sum test. For categorical variables, percentages are presented in parentheses, and Pearson's chi‐squared test or Fisher's exact test is employed. A *p*‐value of <0.05 is considered to indicate statistical significance.

Abbreviations: APOS, acute postoperative seizure; EEG, electroencephalogram; FCD, focal cortical dysplasia; LFU, last follow‐up; MCD, malformation of cortical development; MOGHE, mild malformation with oligodendroglial hyperplasia in epilepsy; MRI, magnetic resonance imaging; postsurgical AEDs, postsurgical number of anti‐seizure medicines; presurgical AEDs, presurgical number of anti‐seizure medicines; SEP/MEP, somatosensory evoked potentials/motor evoked potentials.

**FIGURE 4:**
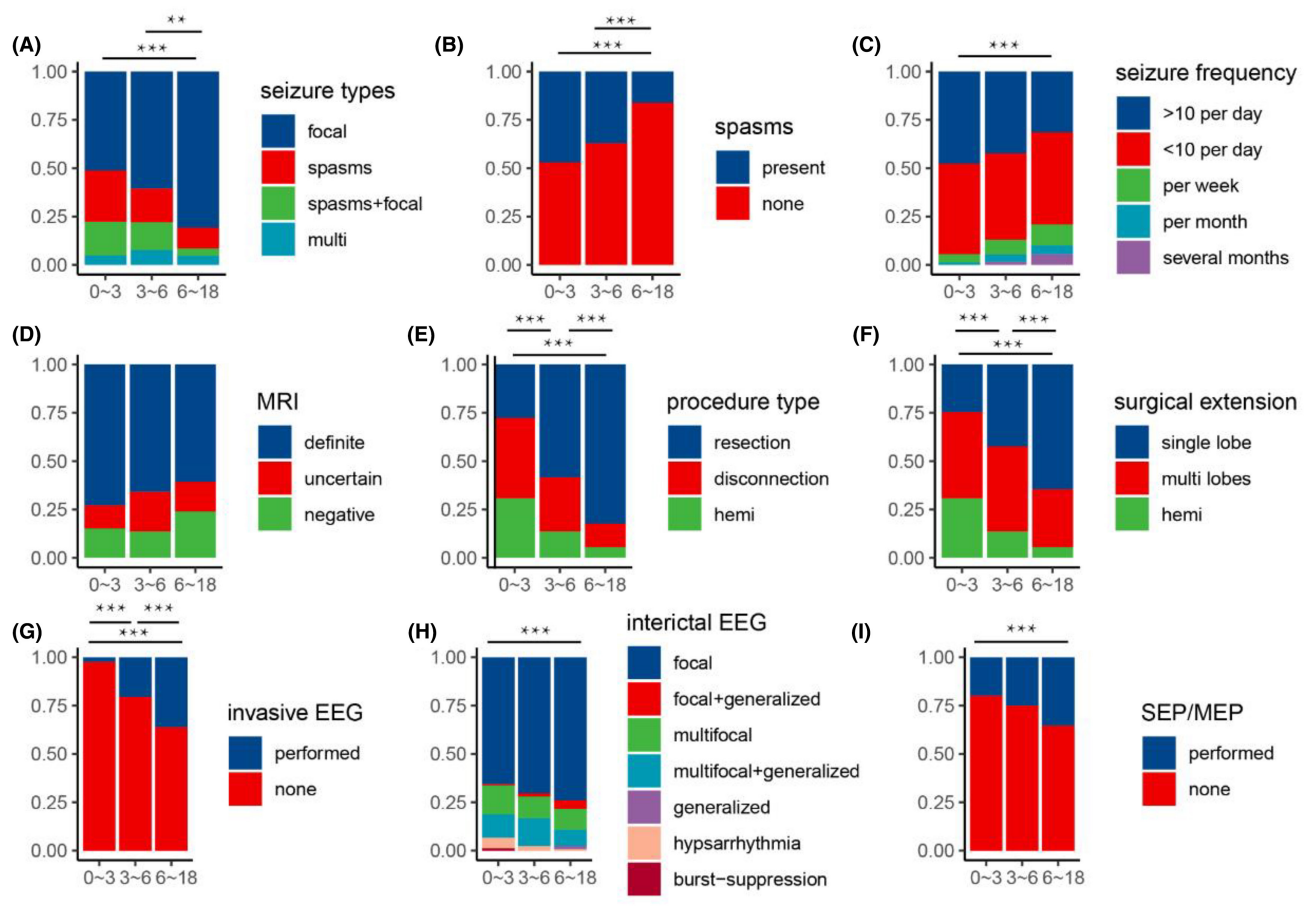
Clinical characteristics of different age groups: (A) seizure types, (B) spasms, (C) siezure frequency, (D) MRI, (E) procedure type, (F) surgical extension, (G) invasive EEG, (H) interictal EEG, (I) SEP/MEP. Except for Figure (D), the other images all demonstrate varying degrees or combinations of statistically significant differences among the three age groups. **p < 0.01; ***p < 0.001.

For the variables with significant differences, pairwise comparisons within each group were conducted, and the results are depicted in the figures (Figure 4). Interestingly, when conducting pairwise comparisons between different age groups, there were no significant differences in MRI findings, contrary of the univariate analysis results.

## 4 DISCUSSION

MCD often leads to structural changes in the central nervous system and is frequently associated with epileptic manifestations. In the early stages of seizures, only 17% of patients achieved seizure freedom for at least 1 year despite pharmacological intervention.^17^ However, with long‐term observation, these cases often progressed to DRE.^4^ Surgical treatment, involving the removal or isolation of the epileptogenic lesion, is considered the most effective way to terminate epilepsy. However, locating and defining the surgical resection extension for certain MCD lesions poses significant challenges, making surgical treatment more difficult compared to other etiologies such as hippocampal sclerosis and developmental tumors.^1^, ^18^

The mechanisms and prognosis of epilepsy show significant differences between adults and children.^19^ Therefore, we believe that the clinical characteristics and treatment strategies of MCD vary across different age groups, exhibiting dynamic changes over time.

Since our center was established in 2014, we have completed over 1000 pediatric epilepsy surgeries, with MCD accounting for the majority at approximately 60% or more. This study included 428 pediatric patients with MCD, and in the follow‐up of at least 3 years, the seizure‐free rate was 81.3%. Compared to previous literature reporting an average postoperative seizure‐free rate of 62% for MCD treatment, our results are quite satisfactory.^20^

We summarized the surgical treatment outcomes of 113 cases of DRE in children under 3 years old at a single center. The seizure‐free rate at the last follow‐up was 86.7%, slightly lower than the 88.6% seizure‐free rate in the present study for children under 3 years old.^21^ This difference may be attributed to the shorter follow‐up time or the closer surgical timing of the cases. Gradually enriched evaluation and surgical experience have improved the postoperative seizure‐free rate. The use of high‐field MRI, FDG‐PET, PET‐MRI fusion, widespread application of SEEG, protective measures for sensory‐motor cortex during surgery, advances in preoperative assessment, and evolving assessment and surgery strategy have significantly increased the postoperative seizure‐free rate compared to a decade ago.

In univariate analysis, APOS was found to be correlated with prognosis, consistent with several previous studies.^16^, ^22^, ^23^ However, in multivariate analysis, APOS did not emerge as a predictive factor for seizure prognosis. Most literature reports on APOS lack descriptions of seizure types. In our study, we classified seizure forms based on whether they were consistent with preoperative habitual seizures, finding a correlation between habitual seizures and prognosis. Therefore, during the perioperative period, if APOS was present, physicians need to rule out the possibility of seizures caused by surgical stimulation of the lesion's surrounding cortex or edema of surrounding brain tissue. This helped determine whether it is consistent with preoperative habitual seizures. Even though the presence of habitual seizure predicts a poor prognosis, 12 out of 18 children with habitual seizure achieved seizure freedom. This suggests that these temporary seizures did not hinder the overall success of the surgery and may resolve as the brain healed and stabilized postoperatively. Patients with negative MRI results had a poorer prognosis.^24^ However, the use of high‐field MRI and specific seizure sequences assisted in detecting MCD lesions, reducing the occurrence of MRI negativity.^25^ We believe that identifying MCD lesions through MRI requires a substantial amount of image reading and clinical experience, which is a highly subjective task. Therefore, in preoperative assessments, careful examination of MRI layer by layer is necessary to discover lesions and reduced the MRI negativity rate. The higher incidence of MRI‐negative findings in this study was initially observed because a comprehensive approach incorporating EEG and FDG‐PET results was not used uniformly before presurgical evaluation. When EEG and FDG‐PET findings were integrated, MRI were reviewed by presurgical evaluation members and lesions were subsequently identifiable on MRI for all patients. Invasive EEG was generally applied to patients with unclear lesion location or extent. An unpublished study in our center included 102 children who underwent scheduled craniotomy for lesion removal after SEEG implantation, resulting in a seizure‐free rate of 69.6%. Therefore, the prognosis for children using invasive EEG was worse than for those directly undergoing curative treatment, but the surgical outcomes were still satisfactory.

Surgery for FCD1 type presented certain difficulties and poor outcomes mainly due to the extensive lesion range and difficulty in detection on MRI, as its pathological changes involved only disorganized alterations in cortical cells.^26^, ^27^ FCD3 type, defined as FCD1 dyslamination pathology with other types of lesions, was easier to detect on MRI but required a larger resection extension than the accompanying lesion to ensure complete removal of the layered abnormal cortex around it.^14^ In our cohort, the seizure‐free rates for FCD1, MOGHE, and other MCDs were 17.5%, 14.3%, and 23.6%, respectively, significantly lower than the other two pathological types. FCD2 had a better prognosis, possibly because it was more apparent on EEG and MRI, had a clear boundary, and often involved a relatively limited brain lobe, making it easier to determine the resection scope.^28^

Although literature reports suggested that one‐third of FCD2 types were MRI‐negative, this may be attributed to the use of inadequate epilepsy sequences and low‐field MRI.^29^ In our previous retrospective study on surgical treatment for DRE in children under 3 years old, MCD, although the most common cause, posed significant challenges in lesion localization and determination of lesion scope. Moreover, in cases of seizure recurrence, MCD had the highest proportion, indicating ongoing difficulties in the surgical treatment of MCD.^21^

We conducted a simple statistical analysis of the number of AEDs used by the children just before surgery, finding a correlation with prognosis, serving as a predictive factor. This has not been reported in previous literature, but it had certain limitations since many children had tried multiple AEDs before surgery, discontinuing or adding more due to inefficacy. If all types of drugs used and their changes before surgery were collected, it would provide a more representative result. However, the number of postoperative used AEDs correlated with prognosis, as expected, so it was not included in the multifactor analysis. In the multivariate logistic regression analysis, surgical age, as well as preoperative AEDs, emerged as predictive factors, with the model's AUC >0.7, indicating satisfactory predictive ability.

In adults with long‐term seizure suffering, epilepsy can still lead to synaptic reshaping, altering the seizure network.^30^ Additionally, a considerable number of MCD patients exhibited a seizure pattern as a network involving anatomically distant structures or affecting both hemispheres.^31^ We hypothesized that if MCD persisted for a long time without timely surgical treatment, abnormal discharges from the lesion might lead to electrophysiological abnormalities in the surrounding cortex or even contralateral hemisphere, forming a new epileptic network which could lead to recurrence. However, there may be no apparent structural changes, making it undetectable by MRI. Therefore, even if the structurally defined epileptic focus is removed, the electrophysiologically epileptogenic lesion may persist, posing a significant challenge in the surgical treatment of MCD.

The development of the central nervous system in children is a continuous process, particularly before the age of 6, encompassing events such as myelination, which often completes around age 3 or even up to 60 months.^32^, ^33^ By age 6, language skills were typically fully developed. Accordingly, we categorized all cases into three age groups. Younger children exhibit an immature neural network and a higher frequency of seizure compared to older children. Clinically, spasm seizures are more common in younger patients. The majority of asymmetric spasm seizures may indicate the likelihood of structural etiology.^34^ However, the prognosis for children with spasms was not significantly different from other types of seizures, consistent with our previous research. We believe that structural changes are critical to surgical outcomes, and the presence of spasms should not preclude surgical intervention.^35^ Younger children (0–3 years) tended to have an earlier onset of epilepsy and a shorter duration of the disease before surgery (Figure 3). These factors likely contribute to the higher rates of seizure freedom observed in this age group, as early intervention may prevent the establishment of more complex epileptic networks. Regarding MRI findings, older children tended to have less prominent MCD lesions, possibly due to earlier surgical intervention if lesions were significant at a younger age, whether at other hospitals or at our clinic. However, this phenomenon was not significant in pairwise comparisons among the three age groups. As a tertiary epilepsy center, we utilize extensive clinical experience and various invasive EEG and/or SEP/MEP techniques to ultimately locate and excise the lesion which was regarded as suspected or negative in MRI, achieving seizure‐free outcomes of the older children. Younger patients often undergo more extensive resections or disconnections. Recently, for children with MCD, total lesionectomy or broader resections tended to yield better results.^36^, ^37^ This can also explain the predictive factor of surgical age, as younger children with more plastic neural systems often underwent more aggressive lesion resections, leading to better surgical prognoses. In older patients, functional preservation became a priority, hence the increased application of SEEG and evoked potential monitoring. Interictal EEG focal discharges have a certain value in lesion localization, but diverse and extensive discharges in younger children make preoperative assessment challenging. The younger the patient at the time of surgery, the better the prognosis, especially for MCD‐related DRE.^38^ Clinicians should recommend early surgical intervention by minimizing the duration of epilepsy to halt seizures.^39^ In the perioperative period, habitual seizures were more common in older children due to their more developed and mature nervous systems.

Among all patients, the longest duration of illness reached 14.3 years. The reason for such prolonged periods without effective treatment was that the families of children with epilepsy and some doctors in primary care hospitals, lacked a full understanding that DRE caused by structural epileptogenic lesions can be cured through surgical treatment. This lack of awareness has led to delays in diagnosis and treatment, hindering the normal development of the affected children and imposing significant additional economic burdens on their families. The initial seizure onset caused by MCD rarely occurred after reaching adulthood.^40^ Therefore, after the onset of epilepsy in minors, it was imperative to quickly ascertain the cause of epilepsy. If MRI showed signs of MCD, preparations for surgical treatment should be made promptly to shorten the duration of the disease and minimize harm to the child and their family.

## 5 CONCLUSION

MCD‐related DRE can be effectively managed through surgical intervention. Factors such as surgical age, MRI findings, invasive EEG, pathology, APOS, and the number of AEDs before and after surgery are related to prognosis. However, only surgical age and the number of preoperative AEDs are seizure‐outcome predictive factors. The younger the age and the fewer preoperative AEDs taken preoperatively, the better the prognosis. Children of different age groups vary in their disease duration, age of onset, MRI findings, seizure types, invasive EEG, SEP/MEP, surgical approaches, extent of resection, and perioperative habitual seizures.

## AUTHOR CONTRIBUTIONS

LC designed this study. LC, XL, and YJ revised the manuscript. HY analyzed the data and drafted and revised the manuscript. YS collected the data. QL, CL, and YW helped to select the patients. TJ and SW helped to interpret the EEG data. All the authors contributed to the article and approved the submitted version.

## CONFLICT OF INTEREST STATEMENT

Neither of the authors has any conflict of interest to disclose.

## Supporting information


Data S1:

## Data Availability

The raw data supporting the conclusions of this article can be obtained from the authors upon reasonable request.
