# Supplementary material for: Clinical characteristics and post‐operative outcomes in children with malformation of cortical development related drug‐resistant epilepsy: 428 cases in one pediatric epilepsy center
Source: CNS Neurosci Ther. 2024 Sep 4;30(9):e70031. doi: 10.1111/cns.70031 (PMC11374691; doi:10.1111/cns.70031)
Supplement: Supplementary file 1 — Data S1: [file CNS-30-e70031-s001.docx]

Table 1 Repeated procedures and seizure outcomes

| **ID** | **Sex** | **Surgery age** | **Side** | **Initial surgery** | **Second surgery** | **Third surgery** | **Seizure outcomes** |
| --- | --- | --- | --- | --- | --- | --- | --- |
| 2 | female | 1.36 | R | Frontal resection | Sub-total hemispherotomy |  | I |
| 16 | male | 2.72 | L | Frontal and insular resection | Enlargement resection |  | I |
| 34 | male | 4.54 | L | Temporal and occipital resection | TPO disconnection |  | III |
| 35 | male | 3.79 | R | Frontal and insular resection | Temporal resection |  | III |
| 56 | male | 5.33 | R | Hemispherotomy | Enlargement resection | Hemispherotomy | I |
| 66 | male | 0.84 | R | Hemispherotomy | Hemispherotomy |  | I |
| 80 | male | 1.24 | L | Hemispherotomy | Re-hemispherotomy |  | I |
| 94 | female | 4.41 | L | TO disconnection | Amygdalohippocampectomy |  | I |
| 99 | female | 4.51 | L | Parietal resection | Hemispherotomy |  | I |
| 100 | male | 1.15 | L | TPO disconnection | Enlargement resection | VNS | IV |
| 109 | male | 15.29 | R | Parietal resection | TPO disconnection |  | IV |
| 112 | male | 2.38 | R | Frontal resection | Enlargement resection |  | I |
| 121 | male | 7.68 | L | Temporal resection | Enlargement resection |  | II |
| 126 | female | 3.68 | R | Frontal and parietal resection | Hemispherotomy |  | I |
| 143 | female | 4.54 | L | Temporal， parietal and insular resection | Enlargement resection |  | I |
| 162 | male | 8.44 | R | Frontal and insular resection | Enlargement resection |  | IV |
| 165 | female | 2.00 | L | Frontal resection | Hemispherotomy |  | I |
| 186 | female | 3.77 | R | Frontal disconnection and parietal resection | Hemispherotomy |  | I |
| 208 | male | 4.98 | L | Frontal and parietal resection | Frontal disconnection |  | I |
| 215 | female | 0.75 | L | Frontal disconnection | Hemispherotomy |  | I |
| 229 | female | 1.10 | L | Temporal， parietal， temporal and insular resection | CC | Hemispherotomy | I |
| 284 | female | 2.73 | R | Frontal disconnection，parietal and insular resection | Hemispherotomy |  | I |
| 299 | female | 2.63 | L | Parietal resection | Enlargement resection | Enlargement resection | IV |
| 320 | male | 3.14 | L | Temporal， parietal， temporal and insular resection | Enlargement resection | VNS | IV |
| 329 | male | 1.73 | L | Parietal and occipital resection | TPO disconnection |  | I |
| 330 | female | 4.43 | L | Frontal and central cortex resection | TPO disconnection |  | I |
| 344 | female | 3.09 | R | TPO disconnection | Sub-total hemispherotomy |  | I |
| 348 | male | 6.24 | L | Frontal resection | Contralateral parietal rescetion |  | I |
| 370 | male | 3.65 | L | Frontal resection | Enlargement resection | Enlargement resection | I |
